# HIV DNA and transcription before and after ART in natural controllers compared to noncontrollers

**DOI:** 10.1128/jvi.00482-26

**Published:** 2026-08-24

**Authors:** Sun Jin Kim, Julie Janssens, Cordelia Isbell, Adam Wedrychowski, Alton Barbehenn, Rebecca Hoh, Michael J. Peluso, Sulggi A. Lee, Satish K. Pillai, Timothy J. Henrich, Nadia R. Roan, Steven G. Deeks, Steven A. Yukl

**Affiliations:** 1 Department of Medicine, University of California, San Francisco (UCSF) 8785, San Francisco, California, USA; 2 Vitalant Research Institute 166672, San Francisco, California, USA; 3 Department of Urology, University of California, San Francisco (UCSF) 8785, San Francisco, California, USA; 4 Gladstone Institutes 40292, San Francisco, California, USA; Icahn School of Medicine at Mount Sinai, New York, New York, USA

**Keywords:** HIV, HIV-1, controller, RNA, transcription, DNA, provirus

## Abstract

HIV-1 natural controllers can limit plasma HIV RNA levels in the absence of antiretroviral therapy (ART). Although controllers have less HIV DNA, it remains unclear how HIV transcription differs from noncontrollers, and whether ART further reduces HIV DNA or RNA in controllers. To address these questions, we quantified different HIV transcripts and HIV DNA regions/proviruses in longitudinal blood samples collected before ART and up to 3–5 years on suppressive ART from 10 controllers (5 “elite” and 5 viremic) and compared them to ART-suppressed noncontrollers. Compared to noncontrollers, controllers exhibited lower levels of most HIV DNA regions (but not intact proviruses) and lower total levels of initiated and completed HIV RNA, but paradoxically higher levels per provirus of initiated and 5′ elongated HIV transcripts. Controllers also showed a lower ratio of completed/elongated HIV RNA. After ART, controllers showed lower levels of multiply spliced HIV RNA and a lower ratio of multiply spliced/completed HIV RNA. These results suggest that natural controllers limit HIV infection frequency and selectively restrict HIV transcript completion and splicing. ART treatment of controllers led to further increases in CD4 counts and decreases in HIV DNA and some HIV transcripts, supporting the potential for clinical benefits in this population.

**IMPORTANCE:**

Natural HIV controllers can suppress HIV-1 replication without antiretroviral therapy (ART) and serve as a model for an HIV functional cure, but the mechanisms are unclear. We found that natural control was associated with mechanisms that reduce levels of total HIV DNA and the HIV promoter regions to below the levels seen in ART-treated noncontrollers (NC), resulting in lower total levels of most HIV transcripts. When corrected for HIV DNA levels, natural controllers exhibited higher levels per provirus of short, prematurely terminated HIV transcripts but lower proportions of completed and spliced HIV transcripts. These findings suggest that the mechanisms of natural control do not only limit HIV infection frequency, but specifically limit production or increase clearance of infected cells making completed and spliced HIV transcripts. ART treatment of controllers led to further increases in CD4^+^ T-cell counts and decreases in HIV DNA and RNA, suggesting that ART should be offered to controllers.

## INTRODUCTION

HIV “controllers” (also called natural controllers or suppressors) constitute a rare subset of people living with HIV (PWH) who are able to limit HIV-1 replication without antiretroviral therapy (ART), resulting in low or undetectable levels of HIV RNA in the plasma (viral load). Viremic controllers can limit viral loads to less than a few thousand copies/mL, while “elite” controllers (EC) are able to suppress viral loads to below the approved detection limits of clinical assays. EC represent <1% of all PWH and have been studied as a model for HIV functional cure or remission (1, 2). EC are a heterogeneous group. Some EC maintain virologic control for long periods (persistent controllers), while others eventually lose virologic control (transient controllers) and/or immunologic control and may progress to AIDS (2, 3).

The mechanisms of natural control are not fully understood, but differences in adaptive and/or innate immune responses may contribute to control. EC are more likely to have “protective” HLA class I alleles and often exhibit strong HIV-specific CD8^+^ (and sometimes CD4^+^) T-cell responses (1, 4). Other factors that may contribute to control include decreased susceptibility of CD4^+^ T cells to HIV infection, higher levels of HIV-specific memory B cells, greater affinity and polyfunctional activity (including ADCC) of HIV-specific antibodies, differences in NK cell phenotypes and functions, decreased susceptibility of dendritic cells (DCs) to infection, and increased antiviral activity of myeloid DC (4).

Reduced viral fitness or gene function may also contribute to some cases of natural control, including deletions in the nef/3′LTR region (5–8) and reduced function of the Gag, Pol (9), and Env proteins (10). Although infectious virus or intact HIV DNA could not be detected from a subset of EC (11, 12), many publications suggest that EC harbor infectious proviruses (11, 13–21).

Multiple studies have reported that controllers have fewer infected cells or less HIV DNA than ART-suppressed PWH. Two studies found that EC had a lower frequency of cells that can be induced by activation to produce virions or infectious HIV (11, 18), while one study found no difference from ART-suppressed PWH (20). Although one study reported no difference in total HIV DNA levels (22)*,* more studies found that EC had lower levels of total (12, 23–26)*,* integrated (22)*,* defective (20, 25), and intact HIV DNA (12, 20, 24, 25). Two studies reported that the ratio of intact/total HIV DNA did not differ between EC and ART-suppressed PWH (20, 24)*,* while one study found that the ratio was higher in EC (12). Nevertheless, intact HIV DNA could not be detected in a subset of EC (10, 12, 25).

Elite control has also been associated with qualitative differences in HIV reservoir diversity, clonality, and integration sites. Compared to ART-suppressed PWH, proviruses from EC show lower diversity and more clonality (12). In EC, a larger proportion of intact proviruses are integrated into regions that are farther from actively transcribed chromatin and enriched for markers of heterochromatin (12). Similar findings were found to distinguish permanent controllers from transient controllers (25, 27). These studies suggest that elite control is associated with “block and lock” mechanisms in which intact proviruses are silenced by integration into repressive chromatin regions.

However, few studies have measured cell-associated HIV transcripts (CA-HIV RNA) in controllers, and only unspliced (gag or pol) HIV RNA has been reported. The frequency of EC in whom unspliced HIV RNA could be detected has varied considerably, from 0/3 in “exceptional elite controllers” (28) to 2/10 (29)*,* 1/2 (30), 5/9 (20), and 25/29 in EC (31). Four studies reported that levels of unspliced HIV RNA were lower in EC than ART-suppressed PWH (12, 20, 29, 30). However, most studies did not normalize HIV RNA levels to the number of proviruses or infected cells, so it is not clear whether the lower total levels of HIV RNA in EC result from fewer infected cells and/or less transcription per infected cell. One study reported that EC had lower levels of HIV RNA per intact HIV DNA than ART-suppressed PWH (12)*,* while a second study found no difference in the ratio of HIV RNA to total HIV DNA in the rectum (26). A third study, which was performed in viremic controllers (VL < 2,000), reported that viral control may be related to having fewer infected cells rather than proviral silencing (32). Therefore, it is unclear whether natural control is associated with lower levels of HIV transcription initiation per infected cell, as might be expected from preferential integration into heterochromatin. Moreover, no prior studies have investigated whether elite control is associated with blocks to HIV transcriptional elongation, completion, or splicing, which would affect HIV replication and are likely mechanisms underlying latent HIV infection in the blood and tissues (33–35).

Finally, it is unclear whether the administration of ART would result in further reductions in the HIV reservoir or different HIV transcripts that may ultimately result in clinical benefit to controllers. Two published studies have analyzed the effect of ART on EC. In one study in which 16 controllers were given ART for 24 weeks, no significant decrease was detected in total HIV DNA or CA HIV RNA in PBMC, while significant decreases were observed in plasma HIV RNA, integrated HIV DNA, CA HIV RNA in rectal cells, and T-cell activation in blood and rectum (36). In the second study, in which three EC and one controller were treated with ART for 9 months, no change was detected in total HIV DNA in blood, but decreases were observed in the viral outgrowth assay (3/4) and T-cell activation (19). It remains unclear whether ART would reduce HIV reservoirs and transcripts, or increase CD4 counts, when given for longer than 6–9 months.

We hypothesized that ART-naïve natural controllers would differ from ART-suppressed PWH in the blocks to various stages of HIV transcription, and that ART would reduce levels of intact proviruses and some HIV transcripts (completed and spliced). To test these hypotheses, we measured levels of various HIV DNA regions/proviruses and HIV transcripts in longitudinal blood samples obtained from 10 natural controllers (5 EC and 5 viremic controllers) before ART (T1) and one to two additional time points (T2 and T3) covering up to 3–5 years on suppressive ART, and we compared these HIV levels to those in chronic-treated, ART-suppressed noncontrollers (NC).

## MATERIALS AND METHODS

### Study participants

The study participants included 10 natural controllers from the UCSF SCOPE HIV cohort (Table S1). The study was originally intended to focus on EC, but due to the difficulty in obtaining longitudinal blood samples from before and after ART in any sort of controllers, the inclusion criteria were modified to include both viremic controllers and EC. Blood samples had been obtained prior to ART initiation (T1) and up to two time points (T2 and T3) on suppressive ART (median years after ART start: T2 = 1.4 and T3 = 4.5). Samples were available from all 10 participants at T1 and T2, while 7 of the 10 participants also had samples available from T3. Blood from each visit was used to isolate peripheral blood mononuclear cells (PBMCs), which were cryopreserved. All study participants provided written informed consent.

HIV DNA and RNA levels in these controllers at T1 (pre-ART) and T2 (~1.4 years after ART) were compared to those previously measured in ART-suppressed NC who had initiated ART during chronic infection (ART-treated NC) (33, 37, 38). Of the ART-treated NC, HIV RNA levels were available from up to 25 people (including 23 with data on levels of the corresponding HIV DNA regions), and levels of intact and defective proviruses were available from 19 people.

### CD4^+^ T cell isolation and nucleic acid extraction

CD4^+^ T cells were isolated from cryopreserved PBMCs (9.5 × 10^6^ to 1.5 × 10^7^ cells) by immunogenic negative selection using the EasySep Human CD4^+^ T cell Enrichment Kit (StemCell Technologies, Catalog #19052) and counted using a Countess 3 Automated Cell Counter (Invitrogen). Total cellular DNA and RNA were extracted from each sample using TRI Reagent (Molecular Research Center, Inc., Catalog #TR 118). Extracted DNA samples were passed through QIAshredder columns (Qiagen, Catalog #79656) to facilitate encapsulation into droplets during droplet digital PCR (ddPCR) (39). DNA and RNA concentrations were determined using the NanoDrop One microvolume spectrometer (Thermo Scientific).

### Quantification of HIV DNA by ddPCR

Levels of different HIV DNA regions, including the U3–U5 Long Terminal Repeat (LTR), *Trans* Activation Response (TAR) region, R-U5-pre-Gag region, and Pol region, were quantified using ddPCR as described previously (33, 40). Each sample was tested in at least two replicate ddPCR wells with a maximum DNA input of 500 ng per well. The Intact Proviral DNA Assay (IPDA; duplex ddPCR for the HIV Packaging Signal [Psi] and Rev Response Element [RRE] regions) was performed as described previously (40, 41). For the IPDA, each sample was tested in six to eight replicate ddPCR wells with a DNA input of 600–750 ng per well. HIV DNA copies were normalized to cell numbers according to the mass of DNA input per well (calculated from the DNA concentration and input volume), assuming that 1 µg of DNA is equivalent to 160,000 cells. pNL4-3 DNA (NIH-AIDS Reagent Program, Catalog #114) and cellular DNA from non-infected donors were used as positive and negative controls, respectively.

### Reverse transcription and quantification of HIV RNA

Total initiated HIV (TAR) transcripts were quantified by a three-step polyadenylation-RT-ddPCR as described previously (33, 40, 42). HIV 5′ elongated (R-U5-pre-gag), mid-transcribed (pol), completed (U3-polyA), and multiply spliced (tat-rev) transcripts were quantified by RT-ddPCR as described previously (33) except that we used two duplex ddPCR reactions: (i) 5′ elongated (in Fam) and mid-transcribed/Pol (in Vic) HIV RNA and (ii) completed (in Fam) and multiply spliced (in Vic) HIV RNA (40). All ddPCR assays included *in vitro* transcribed HIV and virion RNA standards (33) as positive controls and cellular RNA from non-infected donors as the negative control. The copies of each transcript were normalized to cellular transcription (1 µg of RNA, equivalent to ~10^6^ cells) based on the RNA concentration, the input volume into the RT reaction, and the proportion used for each ddPCR well. To account for possible differences in the frequency of proviral mutations in different regions, and how infection frequency and proviral mutations can change over time, levels of each HIV transcript were also normalized to levels of the same or corresponding HIV DNA region measured from the same cells using the same primers/probe.

### Statistical analysis

The Wilcoxon signed-rank test (two-tailed) was used to compare levels of different HIV DNA regions/proviruses, HIV transcripts, and ratios (HIV RNA/RNA or HIV RNA/DNA) within and between time points. The Mann-Whitney test (two-tailed) was used to compare levels of HIV targets in natural controllers to those measured previously in ART-suppressed NC. For calculating the median and *P* values, samples with no detectable HIV were assigned values that were lower than the lowest measured value: 0.1 copy for HIV DNA or RNA, and ≤0.001 for certain HIV ratios. All statistics were performed using GraphPad Prism (Version 9.5.1). Given the relatively small number of comparisons (maximum of four HIV DNA regions or five HIV transcripts), and the fact that adjustment for multiple comparisons can obscure true differences when the number of samples is small (maximum of 10 controllers), *P* values were not corrected for multiple comparisons.

## RESULTS

### Demographic and clinical characteristics

At T1 (pre-ART), the 10 controllers had a median age of 52 (range: 26–77) and a median CD4 count of 640 cells/µL (range: 427–1,321; Table S1). Nine of the 10 controllers had at least one viral load (VL) that was less than the detection limit of the clinical assay from at least one time point before ART. In 6 of the 10, the VL was <40 copies/mL at the last measurement before the start of ART. In five of these individuals, pre-ART VL was consistently less than the detection limit (except for one to two blips), which would meet one definition of “elite” controllers. The median time from HIV diagnosis to the start of ART was 10.7 years (range: 0.3–32.6), and for 8 of the 10 (deemed “long-term controllers”), this time was at least 5.8 years. All ART regimens included three drugs, including two NRTIs (emtricitabine+ tenofovir in 9/10 and lamivudine + abacavir in 1/10) and either an integrase strand transfer inhibitor (7/10) or the non-nucleoside reverse transcriptase inhibitor rilpivirine (3/10; Table S2).

As expected, the peak pre-ART VL was much lower in controllers than NC (*P* < 0.0001; Table S3). We did not detect statistically significant differences between the controllers at T1 and the ART-suppressed comparators in age, CD4^+^ T-cell counts, or CD4/CD8 ratios (Table S3), although there was a trend toward a lower proportion of male participants in the controllers (*P* = 0.08–0.10; Table S3). For the comparison of defective and intact proviral levels between the controllers at T1 and the 19 ART-suppressed NC, there was a trend toward a higher CD8^+^ T-cell count in the controllers (*P* = 0.054). For the comparison of HIV RNA levels between the ART-treated controllers at T2 and the 25 ART-suppressed NC, there was a trend toward a longer duration of ART treatment in the NC (*P* = 0.10; Table S3). Therefore, we performed an additional comparison with a subset of 11 NC who were more closely matched for time on ART (median: 1.0 years; range: 0.4–2.3 years; *P* = 0.34 for comparison to controllers).

### Lower levels of LTR and pol HIV DNA, but not intact proviruses, in untreated controllers compared to ART-suppressed PWH

Levels of the different HIV DNA regions (TAR, R-U5-pre-gag, pol, and U3–U5) were measured to investigate whether there are differences in the prevalence of mutations in these regions and to help normalize levels of the corresponding HIV transcripts (initiated, elongated, mid-transcribed/unspliced, and completed, respectively). Of note, the TAR and U3–U5 regions are present at both ends of an HIV provirus, while the R-U5-pre-gag and pol assays only detect one region per provirus.

**Fig 1:**
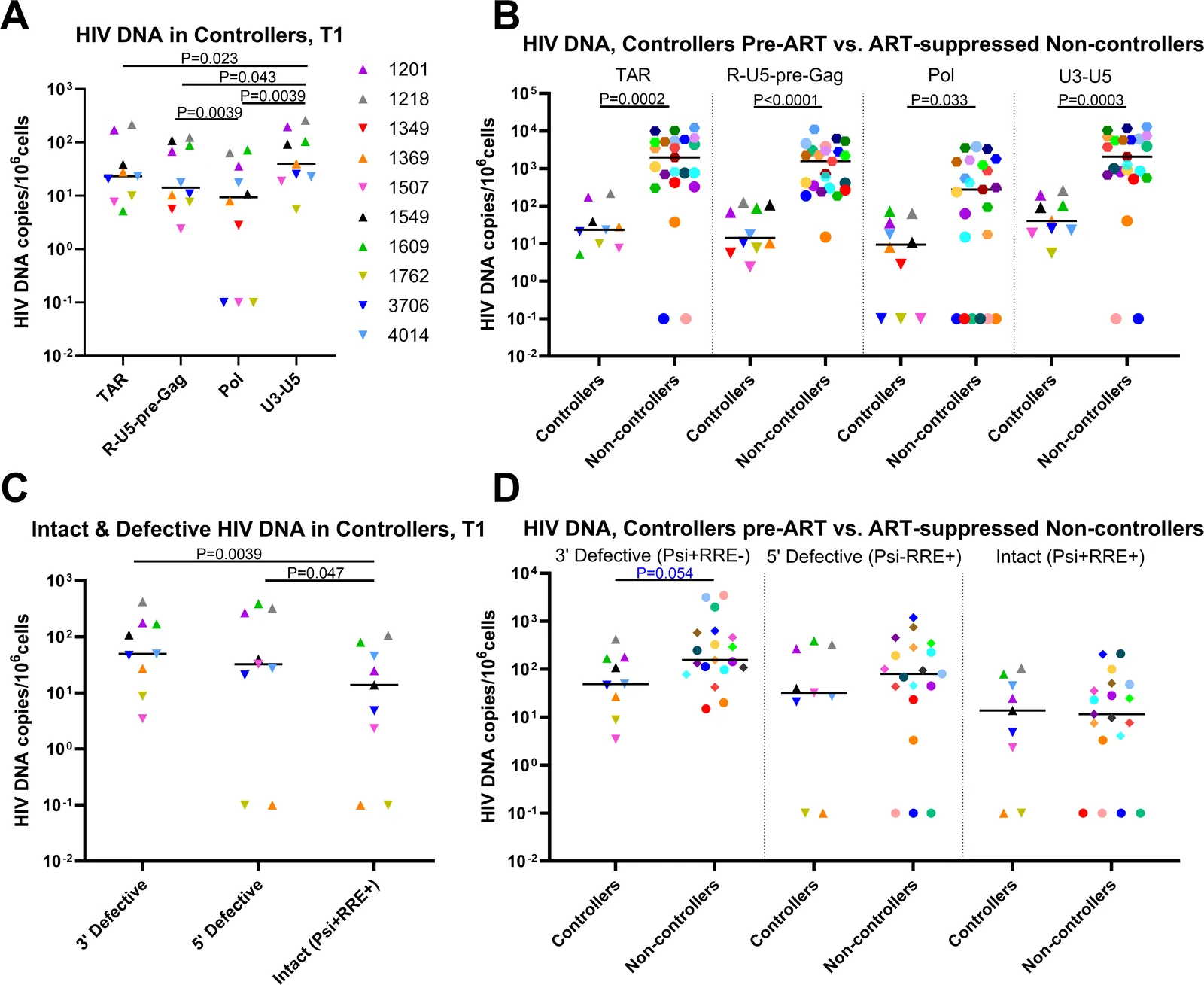
HIV DNA in controllers before ART and comparison to noncontrollers (NC) on suppressive ART. (**A**) Levels of TAR, R-U5-pre-gag, pol, and U3–U5 HIV DNA were measured at T1 (pre-ART) in viremic controllers (triangles) and elite controllers (inverted triangles). (**B**) Comparison between HIV DNA levels in controllers at T1 and ART-suppressed NC from two prior studies (circles or hexagons). (**C**) Levels of 3′ defective (Psi+RRE−), 5′ defective (Psi−RRE+), and intact (Psi+RRE+) HIV proviruses at T1 in controllers. (**D**) Comparison between HIV provirus levels in controllers at T1 and ART-suppressed NC (circles or diamonds). Horizontal lines denote medians. Colors and symbols denote various study participants. Two-tailed *P* values were calculated using the Wilcoxon matched-pairs signed-rank test (**A and C**) or the Mann-Whitney test (**B and D**).

Within untreated controllers, levels of U3–U5 DNA (median: 40 copies/10^6^ cells) were higher than levels of TAR (median: 23; *P* = 0.023), R-U5-pre-gag (median: 14; *P* = 0.043), or pol (median: 9.4; *P* = 0.039) HIV DNA (Fig. 1A). R-U5-pre-gag DNA was also more abundant than pol DNA (*P* = 0.039), possibly reflecting a higher frequency of proviral mutations in pol. Levels of all four HIV DNA regions were much lower in untreated controllers than ART-suppressed NC, with a median fold difference of 85 for TAR (*P* = 0.0002), 111 for R-U5-pre-gag (*P* < 0.0001), 29 for pol (*P* = 0.033), and 52 for U3–U5 DNA (*P* = 0.0003; Fig. 1B). These differences persisted when we limited the comparison of ART-suppressed NC to the five EC (Fig. S1A) or the eight individuals who maintained virologic control (VL < 3,000) for more than 5 years before the start of ART (“long-term controllers;” Fig. S2A).

Since these HIV DNA assays do not distinguish between intact and defective proviruses, we also measured levels of intact and defective proviruses in the controllers using the IPDA. Within controllers at T1, intact (Psi+RRE+) proviruses (median: 13.8 copies/10^6^ cells) were significantly less abundant than either 3′ defective (Psi+RRE−; median 49.3; *P* = 0.0039) or 5′ defective proviruses (Psi−RRE+; median: 32.2; *P* = 0.047; Fig. 1C), and intact proviruses constituted a median of 6.7% of the total detected by the IPDA. Compared to ART-suppressed NC, the controllers tended to have lower levels of 3′ defective proviruses (median: 49 vs 155; *P* = 0.054), while we did not detect significant differences in levels of 5′ defective or intact proviruses (Fig. 1D). Similar findings were observed when we compared ART-suppressed NC to the five EC (Fig. S1B) or the eight long-term controllers (Fig. S2B). When we limited comparisons of HIV DNA regions and IPDA results to the same 10 ART-suppressed NC in whom we had both sets of data, we still observed differences in some HIV DNA regions but not intact HIV DNA (Fig. S3A and B). Overall, these results suggest that the viral control in our study participants is associated with extremely low infection frequencies, but that the landscape of proviruses may differ from ART-suppressed PWH, with the controllers having much lower levels of some HIV DNA regions (particularly the LTR regions involved in HIV transcription) but similar frequencies of intact proviruses.

### Lower total levels of initiated and completed HIV RNA in untreated controllers compared to ART-suppressed PWH

**Fig 2:**
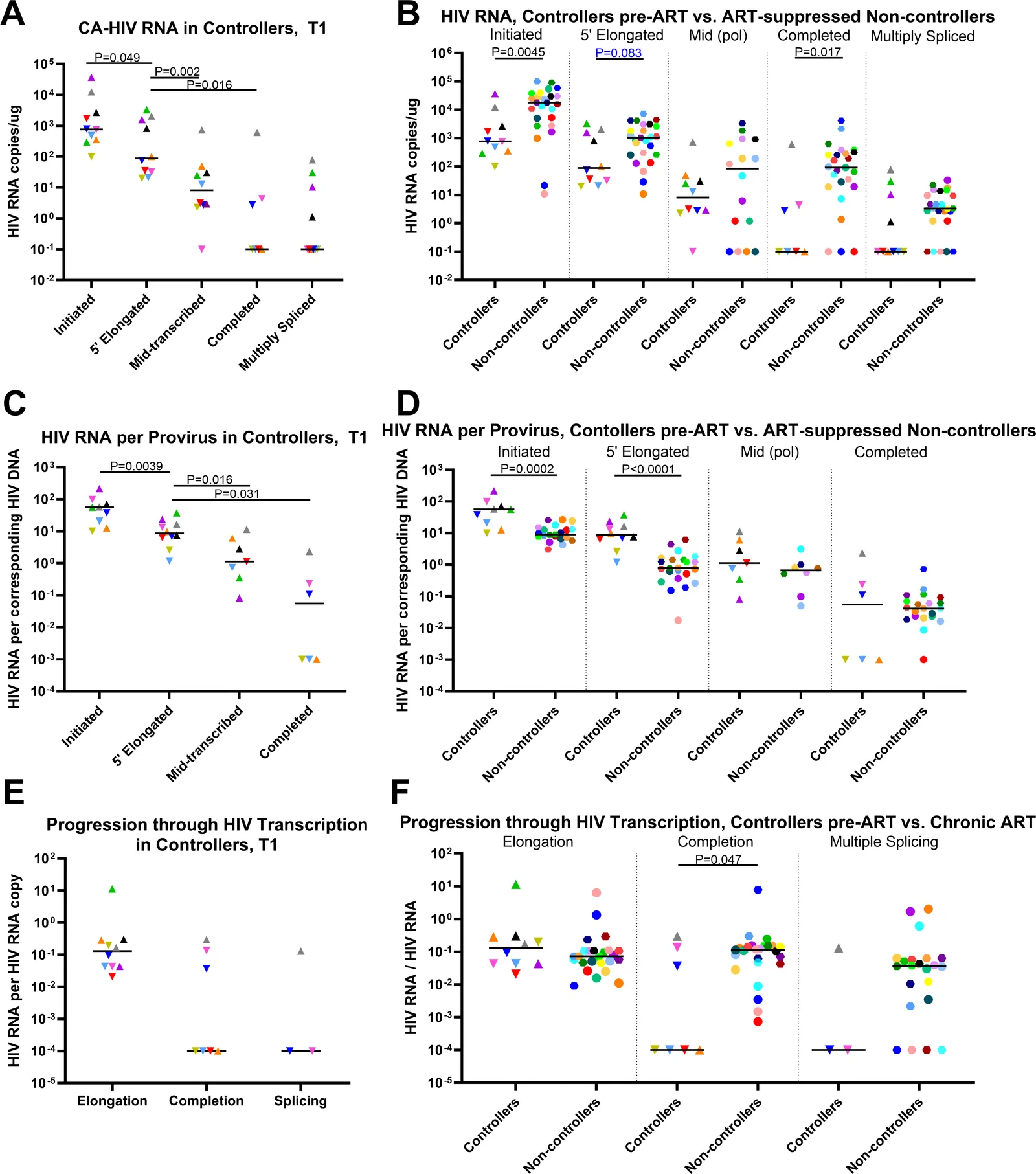
HIV transcription in controllers before ART and comparison to NC on suppressive ART. (**A**) Initiated (TAR), 5′ elongated (R-U5-pre-gag), mid-transcribed (pol), completed (U3-polyA), and multiply spliced (tat-rev) HIV transcripts were measured in viremic controllers (triangles) and EC (inverted triangles) at T1 (before ART). (**B**) Comparison between HIV RNA levels in controllers at T1 and ART-suppressed noncontrollers from two prior studies (circles or hexagons). (**C**) Ratios of initiated, 5′ elongated, mid-transcribed (pol), and completed HIV RNA to the corresponding HIV DNA regions in controllers. (**D**) Comparison between HIV RNA per corresponding HIV DNA in controllers at T1 and ART-suppressed NC (circles or hexagons). (**E**) Progression through stages of HIV transcriptional elongation, completion, and multiple splicing in controllers at T1, as measured by ratios of elongated/initiated, completed/elongated, and multiply spliced/completed HIV RNA. (**F**) Progression through stages of HIV transcription in controllers at T1 vs ART-suppressed NC (circles or hexagons). Horizontal lines denote medians. Colors and shapes denote various study participants. Two-tailed *P* values were calculated using the Wilcoxon matched-pairs signed-rank test (**A,C, and E**) or the Mann-Whitney test (**B, D, and F**).

Within untreated controllers (T1), initiated (TAR) HIV transcripts were more abundant than 5′ elongated (R-U5-pre-gag) HIV transcripts (median elongated/initiated = 0.13; *P* = 0.0039), suggesting a block to HIV transcriptional elongation (Fig. 2A). In addition, 5′ elongated HIV RNA was more abundant than either pol HIV RNA (*P* = 0.002) or completed (U3-polyA) HIV RNA (*P* = 0.016), suggesting a block to HIV transcriptional completion. Completed and multiply spliced (tat-rev) HIV RNA were only detected in 3/10 and 4/10 controllers, respectively. Compared to ART-suppressed NC, the pre-ART samples from controllers showed lower total levels of initiated HIV RNA (median fold difference: 23; *P* = 0.0045), a trend toward lower levels of 5′ elongated HIV RNA (median fold difference: 11.8; *P* = 0.083), and lower levels of completed HIV RNA (median < 1 vs 92 copies/µg; *P* = 0.017; Fig. 2B). When we limited the comparison of ART-suppressed NC to the five EC (Fig. S1C) or the eight long-term controllers (Fig. S2C), both subsets of controllers showed lower total levels of initiated, 5′ elongated, completed, and multiply spliced HIV RNA.

### Higher levels per provirus of initiated and 5′ elongated HIV transcripts, and a lower ratio of completed/elongated HIV RNA, in untreated controllers

To investigate whether differences in HIV RNA levels can be explained by mutations in the HIV DNA, we normalized levels of each HIV RNA to levels of the corresponding HIV DNA region measured from the same cells. Within controllers at T1, levels of initiated HIV RNA/DNA were significantly greater than levels of 5′ elongated HIV RNA/DNA (median: 56.5 vs 8.6 copies/provirus; *P* = 0.0039), and levels of 5′ elongated HIV RNA/DNA were significantly greater than completed HIV RNA/DNA (median: 8.6 vs 0.055 copies/provirus; *P* = 0.031; Fig. 2C). These results suggest that the differences between levels of initiated, elongated, and completed HIV transcripts in controllers are not explained by mutations in the HIV DNA, but may instead reflect blocks to HIV transcriptional elongation and completion.

Compared to ART-suppressed NC, the pre-ART samples from the controllers showed higher levels of initiated HIV RNA/DNA (median: 56.5 vs 8.9 copies/provirus; *P* = 0.0002) and higher levels of 5′ elongated HIV RNA/DNA (median: 8.6 vs 0.8 copies/provirus; *P* < 0.0001), while we detected no difference in completed HIV RNA/DNA (median: 0.055 vs 0.041 copies/provirus; *P* = 0.9; Fig. 2D). Similar findings were observed when the analysis was limited to the five EC (Fig. S1D) or the eight long-term controllers (Fig. S2D). These results suggest the lower total levels of some HIV transcripts in controllers may be driven by lower infection frequency rather than mechanisms that limit HIV transcription initiation per provirus, which was actually higher in controllers.

We also calculated the ratios of one HIV RNA to another to measure progression through the stages of HIV transcriptional elongation (5′ elongated/initiated), completion (completed/elongated), and multiple splicing (multiply spliced/completed). Compared to ART-suppressed NC, the pre-ART samples from the controllers showed a lower extent of HIV transcriptional completion (*P* = 0.047) and a non-significantly lower median level of splicing (*P* = NS), while we did not detect a difference in HIV transcriptional elongation (Fig. 2E and F). Likewise, when the comparison was limited to the subset of five EC, we detected no difference in 5′ elongation (*P* = NS) between EC and ART-suppressed NC, but EC showed lower levels of completion (*P* = 0.019) and a trend toward less multiple splicing (*P* = 0.087; Fig. S1E). The median level of HIV transcriptional completion was also lower in the eight long-term controllers than the ART-suppressed NC, although the difference did not reach significance (*P* = 0.2; Fig. S2E). Overall, these results suggest differences between natural controllers and ART-suppressed PWH in the mechanisms that limit HIV expression, with controllers showing lower total levels of some HIV transcripts (driven mostly by lower levels of HIV DNA), paradoxically higher levels of initiated and elongated HIV transcripts per provirus, and a greater ability to limit HIV transcriptional completion and/or clear cells expressing completed HIV transcripts.

### Increase in CD4^+^ T-cell counts after ART treatment of controllers

**Fig 3:**
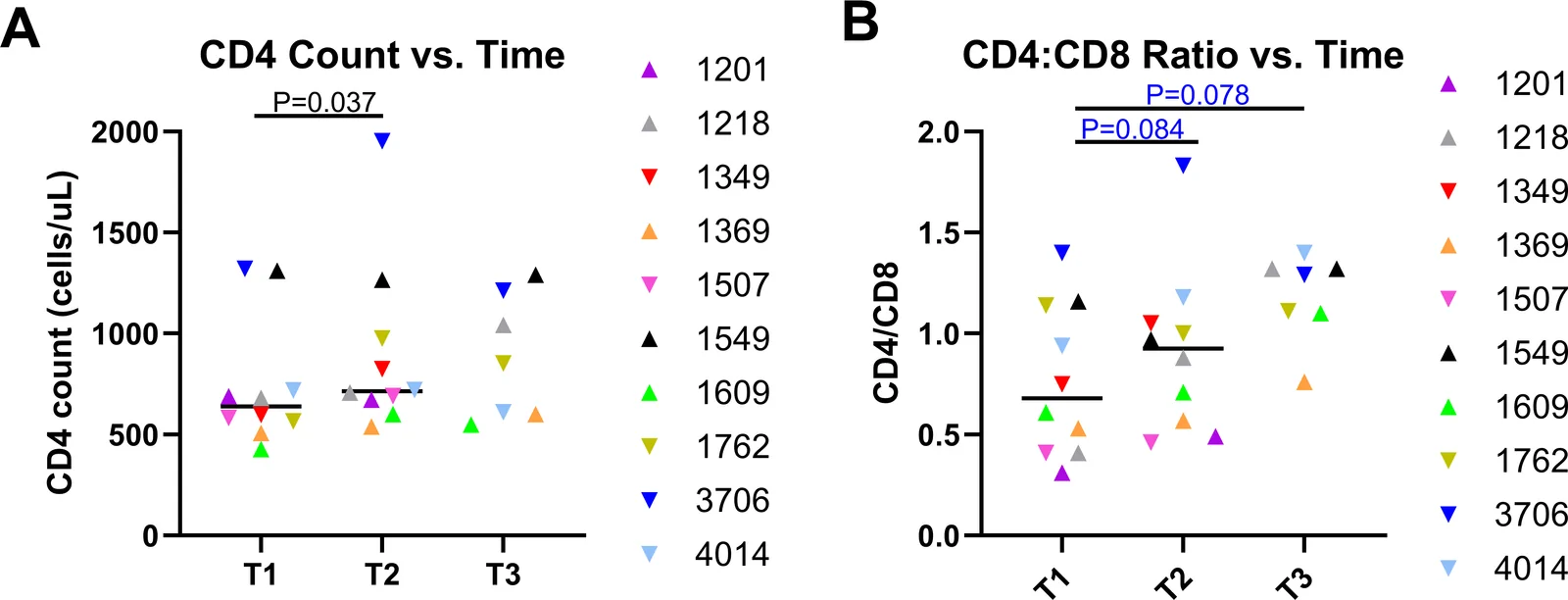
Changes in CD4 counts and CD4:CD8 ratios in controllers after the start of ART. Absolute CD4^+^ T-cell counts (**A**) and the ratio of CD4^+^ to CD8^+^ T cells (**B**) were measured in blood from viremic controllers (triangles) and EC (inverted triangles) before ART (T1) and a median of 1.4 years (T2) and 4.5 years (T3) after the start of ART using clinical assays. Horizontal lines denote medians. Colors denote study participants. Two-tailed *P* values were calculated using the Wilcoxon matched-pairs signed-rank test.

After starting ART, we observed a significant increase in CD4^+^ T-cell counts in controllers from T1 to T2 (median T2-T1 = +70 cells/µL; range: −45 to +631; *P* = 0.037) but no significant change from T2 to T3 (Fig. 3A). In addition, we observed trends toward an increase in the CD4/CD8 ratio in controllers from T1 to T2 (median increase: 0.14; *P* = 0.084) and from T2 to T3 (median increase: 0.27; *P* = 0.078; Fig. 3B). While it would not be possible to detect a statistically significant change in the subset of five EC, they showed a trend toward an increase in CD4^+^ T-cell counts from T1 to T2 (*P* = 0.063; Fig. S4A). Likewise, the subset of eight long-term controllers showed a nonsignificant increase in CD4^+^ T-cell counts from T1 to T2 (*P* = 0.15; Fig. S5A) and trends toward increases in CD4/CD8 ratios from T1 to T2 (*P* = 0.078), T2 to T3 (*P* = 0.063), and T1 to T3 (*P* = 0.063; Fig. S5B). These findings suggest that ART treatment of controllers leads to some immune reconstitution.

### Decreases in all HIV DNA regions and proviruses after ART treatment of controllers

**Fig 4:**
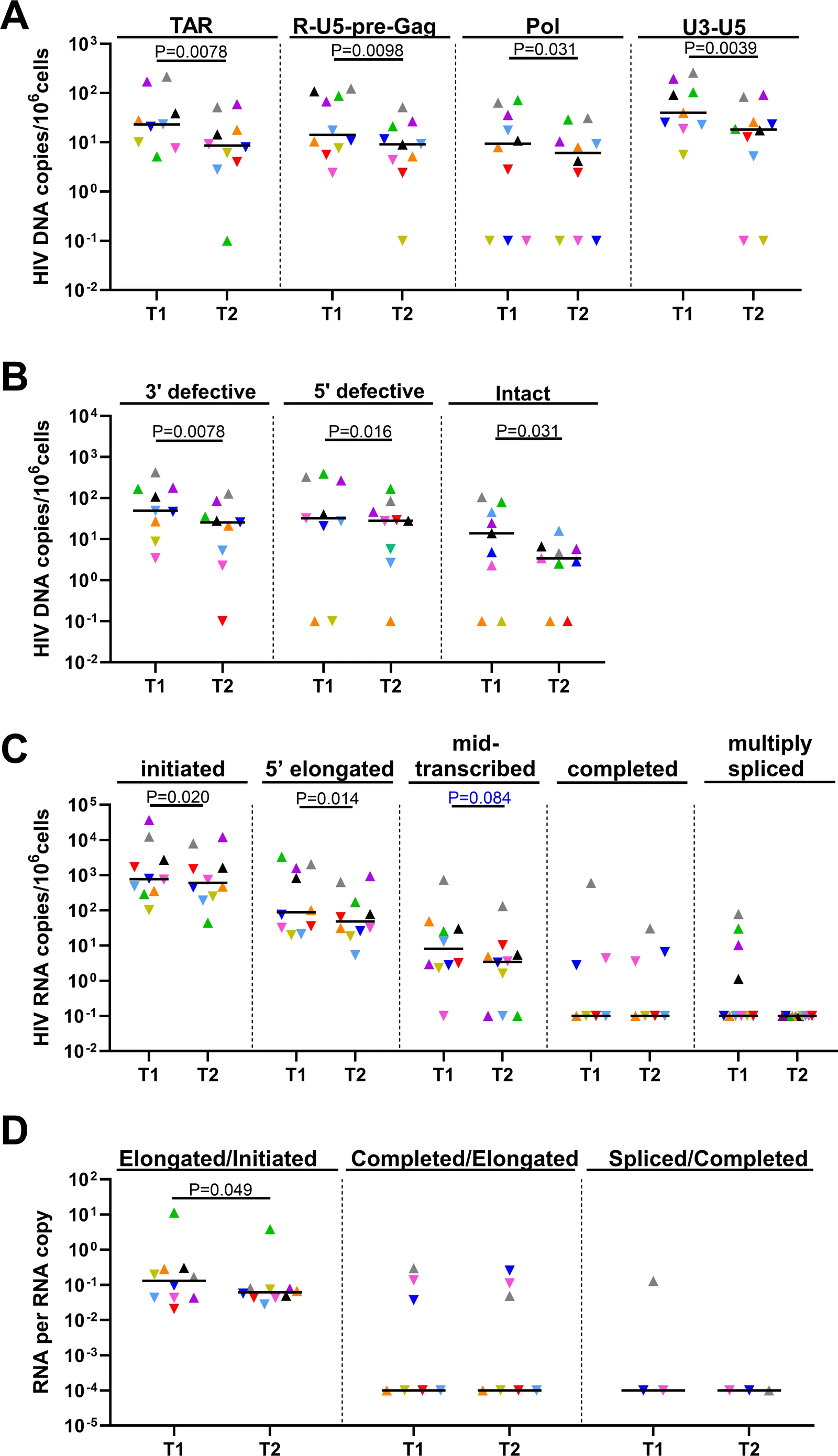
Changes in HIV DNA and HIV transcription in controllers after the start of ART. HIV DNA and HIV transcription were measured in blood from viremic controllers (triangles) and EC (inverted triangles) before ART (T1) and a median of 1.4 years (T2) and 4.5 years (T3) after the start of treatment, including (**A**) Levels of the TAR, R-U5-pre-gag, pol, and U3–U5 HIV DNA regions; (**B**) Levels of 3′ defective (Psi+RRE−), 5′ defective (PsiRRE+), and intact (Psi+RRE+) HIV proviruses; (**C**) Levels of initiated (TAR), 5′ elongated (R-U5-pre-gag), mid-transcribed/unspliced (pol), completed (U3-polyA), and multiply spliced (tat-rev) HIV transcripts; and (**D**) Progression through stages of HIV transcriptional elongation (elongated/initiated RNA), completion (completed/elongated RNA), and multiple splicing (multiply spliced/completed RNA). Horizontal lines denote medians. Colors denote study participants. Two-tailed *P* values were calculated using the Wilcoxon matched-pairs signed-rank test.

From T1 to T2, initiation of ART led to significant decreases in all HIV DNA regions, including TAR (*P* = 0.0078; median T1–T2 = 12.8 copies/10^6^ cells; median T2/T1=0.37), R-U5-pre-gag (*P* = 0.0098; median T1–T2 = 8.4; median T2/T1 = 0.42), pol (*P* = 0.031; median T1–T2 = 6.6; median T2/T1 = 0.45), and U3–U5 DNA (*P* = 0.0039; median T1–T2 = 18.7; median T2/T1 = 0.23; Fig. 4A). In addition, significant decreases were observed in 3′ defective (*P* = 0.0078; median T2–T1 = 61.9 copies/10^6^ cells; median T2/T1 = 0.39), 5′ defective (*P* = 0.016; median T1–T2 = 20; median T2/T1 = 0.35), and intact proviruses (*P* = 0.031; median T1–T2 = 13.1; median T2/T1 = 0.42; Fig. 4B). Within the subset of five EC, median levels of most HIV DNA regions (except pol) and proviruses (except intact) were also lower at T2 than T1 (*P* = NS; Fig. S6A and B). In the subset of eight long-term controllers, levels of all HIV DNA regions and both defective proviruses decreased from T1 to T2 (all *P* < 0.05), and there was a trend toward a decrease in intact proviruses (*P* = 0.063; Fig. S7A and B). These findings suggest clearance of some HIV-infected cells after ART-mediated interruption of ongoing viral replication. From T2 to T3, no additional decreases were observed in any HIV DNA region (Fig. S8A) or type of provirus (Fig. S8B), which could reflect the lower power to detect changes and/or attainment of an equilibrium.

### Decreases in initiated and 5′ elongated HIV RNA after ART treatment of controllers

From T1 to T2, ART led to significant decreases in initiated (*P* = 0.020; median T1–T2 = 270.5 copies/µg RNA; median T2/T1 = 0.62) and 5′ elongated HIV RNA (*P* = 0.014; median T1–T2 = 59.6 copies/µg RNA; median T2/T1 = 0.33; Fig. 4C) and a trend toward a decrease in pol HIV RNA (*P* = 0.084; median T1–T2 = 7.9 copies/µg; median T2/T1 = 0.18). No significant decreases were observed in completed (*P* = 0.75) or multiply spliced HIV RNA (*P* = 0.13), but because these transcripts were detected in such a small proportion of participants at T1, it would have been impossible to detect statistically significant decreases. Moreover, multiply spliced HIV RNA became undetectable in all participants at T2 and T3. Within the subset of five EC, median levels of initiated and 5′ elongated HIV RNA were also lower at T2 than T1 (*P* = NS; Fig. S6C). Likewise, the subset of 8 long-term controllers showed a decrease in initiated and 5′ elongated HIV RNA (both *P*<0.05), and a trend toward decreases in pol (*P* = 0.078) and multiply spliced HIV RNA (*P* = 0.13) from T1 to T2 (Fig. S7C). From T2 to T3, no further decreases were observed in initiated, 5′ elongated, or pol HIV RNA (Fig. S8C).

To investigate whether decreases in HIV RNA levels on ART could be due to decreases in the numbers of infected cells or changes in the frequencies of mutations in different parts of the provirus, we calculated the ratio of each HIV RNA to the corresponding HIV DNA measured at the same time point. The ratio of initiated HIV RNA/DNA (HIV transcription initiation) did not decrease from T1 to T2 and actually tended to increase (*P* = 0.078; Fig. S9A), while no significant change was observed in 5′ elongated, pol, or completed HIV RNA/DNA. Within the subsets of five EC and eight long-term controllers, the median level of initiated HIV RNA/DNA was also higher at T2 than T1 (*P* = NS), but we did not detect significant changes in levels of any HIV RNA/DNA (Fig. S6D and S7D). These results suggest that the decreases from T1 to T2 in total levels of initiated, 5′ elongated, and Pol HIV transcripts were driven largely by the clearance of infected cells on ART. From T2 to T3, we observed a subsequent decrease in initiated HIV RNA/DNA (*P* = 0.013) but no changes in 5′ elongated or pol HIV RNA/DNA (Fig. S9B).

We also calculated the ratios of one HIV RNA to another. From T1 to T2, ART was associated with a reduction in the ratio of elongated/initiated HIV transcripts (from median of 0.13 to 0.06; *P* = 0.049; Fig. 4D), suggesting some decrease in HIV transcriptional elongation and/or preferential clearance of cells making elongated HIV RNA. While no significant changes were observed in the ratios of completed/elongated or multiply spliced/completed HIV RNA, it would have been impossible to detect decreases because of the large proportion of samples with undetectable completed and/or multiply spliced HIV RNA at all time points. From T2 to T3, we observed no further change in the ratio of elongated/initiated HIV RNA (Fig. S8D).

### Lower levels of intact proviruses in ART-treated controllers compared to NC

**Fig 5:**
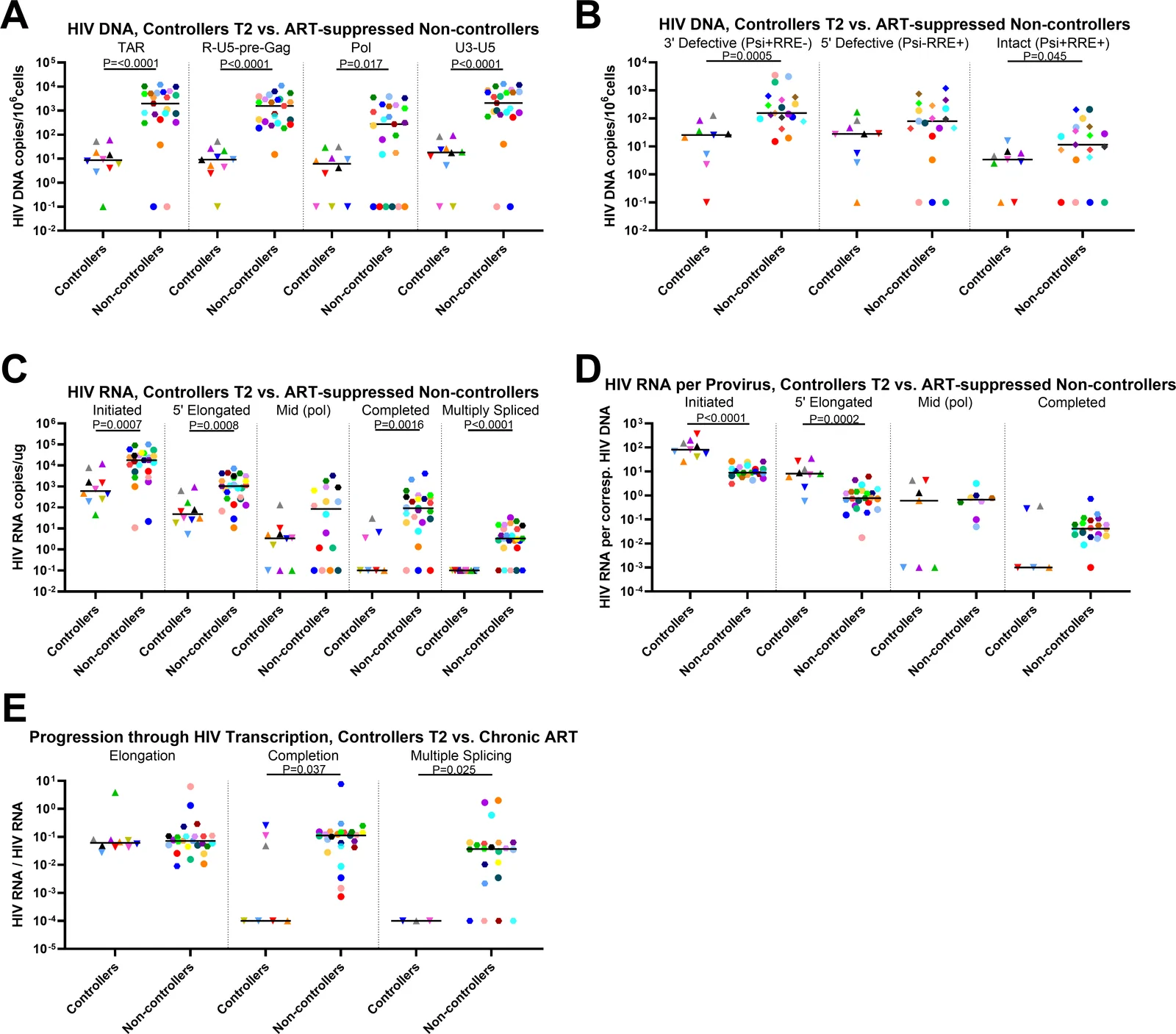
HIV DNA and transcription in ART-suppressed controllers compared to ART-suppressed NC. Levels of HIV DNA (**A and B**), HIV RNA (**C**), HIV RNA per corresponding HIV DNA (**D**), or progression through stages of HIV transcription (**E**) were compared between ART-treated controllers at T2 (triangles denote controllers who were viremic at T1; inverted triangles denote EC) and ART-suppressed NC measured in prior studies (circles, hexagons, or diamonds). Horizontal lines denote medians. Colors and shapes denote study participants. Two-tailed *P* values were calculated using the Mann-Whitney test.

Next, we compared HIV DNA levels in the ART-treated controllers to those observed in ART-treated NC. At T2 (the earliest timepoint post-suppression, with data from all controllers), levels of all four HIV DNA regions were again lower in ART-treated controllers than in NC, with median fold differences of 230 for TAR (*P* < 0.0001), 173 for R-U5-pre-gag (*P* < 0.0001), 44.9 for pol (*P* = 0.017), and 114 for U3–U5 DNA (*P* < 0.0001; Fig. 5A). Compared to ART-suppressed NC, the controllers also had lower levels of 3′ defective proviruses (median: 25.7 vs 155; *P* = 0.0005) and intact proviruses (median: 3.4 vs 11.5; *P* = 0.045), while we did not detect significant differences in levels of 5′ defective proviruses (Fig. 5B).

### Lower levels of multiply spliced HIV RNA and the ratio of multiply spliced/completed HIV RNA in ART-treated controllers compared to NC

Next, we compared HIV RNA levels at T2 in the ART-treated controllers to those observed in ART-treated NC. Compared to 25 ART-suppressed NC, the ART-suppressed controllers showed lower total levels of initiated (median fold difference: 29.5; *P* = 0.0007), 5′ elongated (median fold difference: 21.7; *P* = 0.0008), completed (median < 1 vs 92 copies/µg; *P* = 0.0016), and multiply spliced HIV RNA (median < 1 vs 3.3 copies/µg; *P* < 0.0001), while we did not detect a difference in pol HIV RNA (Fig. 5C). Similar results were obtained when the ART-suppressed controllers were compared to the subset of 11 ART-suppressed NC who were more closely matched for time on ART (median: 1.0 years; *P* = 0.34 for comparison to controllers; Fig. S10).

After normalizing each HIV transcript to the corresponding HIV DNA region, the ART-treated controllers still showed paradoxically higher levels of initiated HIV RNA/DNA (median: 80.5 vs 8.9 copies/provirus; *P* < 0.0001) and 5′ elongated HIV RNA/DNA (median: 8.1 vs 0.8 copies/provirus; *P* = 0.0002) than the 25 NC, while we did not detect differences in pol or completed HIV RNA/DNA (Fig. 5D). The paradoxically higher level of initiated (*P* < 0.0001) and elongated (*P* = 0.0005) HIV RNA/DNA persisted when the controllers were compared to the 11 NC who were more closely matched for ART duration (Fig. S10D). These results suggest that ART did not eliminate the paradoxically higher levels of HIV transcription initiation in controllers, and that the lower levels of HIV DNA in controllers were again contributing to the lower total levels of most HIV transcripts.

We also calculated the ratios of one HIV RNA to another to measure the extent of 5′ elongation (elongated/initiated), completion (completed/elongated), and multiple splicing (multiply spliced/completed). Compared to 25 ART-suppressed NC, the ART-treated controllers still showed a significantly lower extent of HIV transcriptional completion (*P* = 0.037) and now showed a significantly lower level of multiple splicing (*P* = 0.025), while we did not detect a difference in HIV transcriptional elongation (Fig. 5E). When compared to the subset of 11 ART-suppressed NC who were more closely matched for ART duration, the controllers still showed a lower level of HIV multiple splicing (*P* = 0.012; Fig. S10E). Overall, these results suggest that the natural controllers are not better at limiting HIV transcription initiation, but they are better able to reduce the proportion of infected cells in which elongated HIV transcripts have proceeded to completion or splicing, resulting in paradoxically higher levels per provirus of prematurely terminated HIV transcripts.

## DISCUSSION

To investigate the regulation of HIV transcription in natural controllers, we measured levels of different HIV transcripts and HIV DNA regions/proviruses in longitudinal samples obtained from 10 natural controllers (5 elite and 5 viremic) before and after ART, and we compared the results to those observed in ART-suppressed NC. Before ART, controllers showed lower levels of four HIV DNA regions and a trend toward lower levels of 3′ defective proviruses. These results accord with most prior studies targeting other HIV DNA regions (12, 20, 23–26). Possible mechanisms could include those that limit the initial spread of HIV infection (due to either less fit viruses [5–10] and/or enhanced cellular resistance to infection [4]) and/or those that are more effective at clearing infected cells (due to more effective immune responses [4]). Based on the particular regions targeted by our HIV DNA assays, our findings further suggest the ability of natural controllers to limit the number of proviruses with intact LTR (including TAR), 5′ LTR, and Psi regions, which are necessary for HIV transcription and packaging. In contrast to prior published studies, we did not detect a difference in intact HIV proviruses between untreated controllers and ART-treated controllers (12, 20, 24, 25). This discrepancy could reflect differences in our controllers (only half of whom were EC) or the ART-suppressed comparators.

Our study may be the first to quantify HIV transcripts other than unspliced (gag or pol) HIV RNA in controllers. In the ART-naïve controllers at T1, levels of initiated HIV RNA were higher than levels of 5′ elongated HIV RNA, which were higher than levels of completed HIV RNA. This same hierarchy has been observed in blood and tissue cells from ART-suppressed NC (33, 34, 43), where it was explained by reversible blocks at different stages of HIV transcription (33). Differences in levels of these HIV transcripts can also be found in NC before ART, and they increase over time on ART, likely reflecting the effects of immune selection (40). However, while completed and multiply spliced HIV RNA are readily detected in NC before ART (40), and in most people on suppressive ART (33), these transcripts were infrequently detected in natural controllers.

Before ART, controllers expressed lower total levels of the most HIV transcripts than the ART-treated comparators. While we did not detect a difference in unspliced pol HIV RNA, in contrast to some prior studies (12, 20, 29), it should be noted that we had less power to detect a difference in pol (for which we only had data from 16 NC), and the high frequency of mutations in this region could limit the total levels of pol HIV RNA. After normalization to the corresponding HIV DNA regions, HIV RNA/DNA levels were no longer lower in the controllers, suggesting that the lower total levels of some HIV transcripts in controllers were largely driven by a lower frequency of infected cells (most likely) and/or a higher frequency of proviral mutations in the corresponding HIV DNA regions.

Surprisingly, the ratio of initiated HIV RNA/DNA was higher in the untreated controllers (including EC) than the ART-suppressed NC, and the difference persisted after ART treatment. This finding suggests that natural control is not mediated by mechanisms that block HIV transcription initiation. Even in untreated controllers, our data indicate that most initiated HIV transcripts do not extend past the 5′ LTR, and very few of those are complete. These short HIV transcripts may not be recognized by the immune system and may even promote survival of HIV-infected cells by competitive inhibition of Tat, cellular proteins involved in HIV transcriptional elongation (44), or cellular sensors of HIV transcripts, including DDX3 (45). Controllers may also have higher levels of cellular factors that actively block HIV transcription after it starts.

At first glance, the higher level of initiated HIV RNA/DNA in EC seems inconsistent with two prior publications in which elite control has been associated with HIV integration into silent regions of chromatin (12, 25). However, there could be differences between the EC in our study and those in other studies. Moreover, the enrichment for HIV integration into centromeric/satellite DNA or zinc finger genes was primarily observed for intact proviruses, which constitute a small minority (12, 25). We are unable to determine what proportion of the HIV transcripts we measure are being produced by intact or defective proviruses, and it is likely that most of the initiated HIV transcripts are being produced by defective proviruses. However, it is also possible that some of these transcripts are produced by intact proviruses that are not integrated into centromeric/satellite DNA or zinc finger genes, or that integration into these regions does not completely prevent HIV transcription initiation.

This study was also the first to investigate the blocks to HIV transcription in controllers. The ratio of completed/elongated HIV RNA was significantly lower in controllers, suggesting that controllers have a greater block to HIV transcriptional completion and/or immune selection forces that are better at clearing cells producing completed HIV transcripts. It is possible that controllers have better intracellular defenses against HIV, such as RIG-I, which has been reported to recognize genomic HIV RNA (46). Completed HIV transcripts also have greater potential to be translated into HIV proteins, which could be processed to peptides and presented to T cells. Therefore, our findings are also consistent with the enhanced HIV-specific immune responses previously described in controllers (4).

After starting ART, controllers exhibited an increase in absolute CD4+ T-cell counts and a trend toward an increase in the CD4/CD8 ratio. These findings have not been reported in prior studies. One study of 4 ART-treated EC was not powered to detect changes in CD4 counts (19), while another trial of 16 ART-treated controllers found no significant increase in CD4^+^ T-cell counts after 24 weeks (36). The discrepancy from the latter study could be due to differences in the study participants or the methods, particularly the longer follow-up in our study. Our findings suggest that ART facilitates CD4^+^ T-cell reconstitution, which should improve immune function.

Within controllers, ART led to significant decreases in all HIV DNA regions as well as defective and intact proviruses. Although we did not have a control arm, one prior study of untreated controllers showed no significant change in levels of total HIV DNA over ≥20 months of follow-up (31), and the 60–70% decreases that we observed on ART seem too great to be explained by natural processes unrelated to ART. Instead, our findings suggest that ART blocked some new cycles of HIV infection, which limited replenishment of the intact reservoir as well as new defects that can arise after new infection. In addition, ART may have led to more functional immune responses that can clear cells with intact or defective responses, and it may have reduced activation and proliferation of HIV-infected cells. Our findings contrast with the two prior studies, which did not detect a significant decrease in total HIV DNA (19, 36). While the IPDA was not available when those studies were performed, one study found that latent, replication-competent HIV (as measured by viral outgrowth assays) became undetectable in all four controllers after the start of ART (19), which may align with the reduction in intact HIV DNA that we observed. Our findings suggest that ART can significantly reduce HIV reservoirs in controllers, which may confer clinical benefits.

In addition to reducing HIV DNA, ART also reduced the total levels of some HIV transcripts in controllers. Of the two prior studies, one did not assess cell-associated HIV RNA (19), while the other observed reductions in plasma HIV RNA and CA-HIV RNA in the rectum but no change in CA-HIV RNA in the blood (36). The discrepancy from the latter study could relate to differences in the study participants or the methods, especially the time on ART or the HIV RNA assays. The decrease in levels of some HIV transcripts may also confer benefit to controllers, since expression of viral products likely contributes to immune activation and inflammation, which are on average higher in controllers than ART-suppressed PWH. In support of this hypothesis, both of the two prior studies found that ART treatment led to a reduction in T-cell activation in controllers (19, 36).

After ART, controllers had fewer intact proviruses than NC but higher levels of initiated and elongated HIV RNA/DNA, a lower median level of completed HIV RNA/DNA, and lower ratios of completed/elongated and multiply spliced/completed HIV RNA. Together, these findings suggest that the cellular and immune environment of controllers is better at limiting the total number of infected cells, thereby limiting the total production of most HIV transcripts, and does not prevent HIV transcription initiation but is better at limiting the proportion of these transcripts that are completed and spliced. The latter findings were previously observed in post-treatment controllers who interrupted ART (47), suggesting they may be important for both types of controllers. It is possible that controllers differ in the expression of key cellular factors (or possibly viral sequences) that regulate the blocks to HIV completion and splicing (35), and/or that controllers are more effective at clearing those cells in which HIV transcription has proceeded through blocks to completion and splicing. Together, these two mechanisms may preferentially select for cells containing short or incomplete HIV transcripts.

Our study has some limitations. Our controllers were heterogeneous, and only half were EC. While we found significant *P* values, our power to detect other differences may have been limited by the low number of controllers, reflecting the difficulty in obtaining longitudinal pre- and post-ART samples from controllers, as well as the low infection frequencies and division of samples for multiple assays. A proportion of the NC lacked data for all HIV assays, which may have affected comparisons with controllers. Although we assessed for clinical or demographic differences between the controllers and NC, there may have been other differences that were not appreciated. Finally, the timing of the T2 and T3 samples differed somewhat between controllers (Table S2).

Despite these limitations, our study revealed important new information about natural controllers. First, our study provides a more complete picture of the landscape of proviruses and HIV transcripts in controllers before and after ART, and how they differ from NC. Second, our study adds new information about the importance and immunogenicity of different HIV proviruses and transcripts, and how they may be selected by mechanisms of natural control and/or ART. Findings from this study add to a growing body of literature about the importance of completed and multiply spliced HIV transcripts. HIV latency seems to be associated with specific mechanisms that reversibly limit production of these transcripts (33–35), while the ability to clear them (and/or prevent production) seems to be a final common pathway of protective immune responses in natural and post-treatment controllers (47). Third, this study provides additional evidence that controllers may have ongoing replication, which can cause clinical sequelae, and provides new data to support ART treatment for controllers. The ART-mediated reduction in HIV reservoirs, decrease in total HIV transcripts, and increase in CD4^+^ T-cell counts may all lead to improvements in health, suggesting that ART should be offered to natural controllers.

## Data Availability

Demographic and clinical information is shown in Tables S1 through S3. Tabulated data on HIV levels, CD4 counts, and CD4:CD8 ratios are shown in Data Set S1.

## SUPPLEMENTAL MATERIAL

Data Set S1 jvi.00482-26-s0001.xlsx Tabulated data.

Supplemental material jvi.00482-26-s0002.docx Tables S1 to S3; Fig. S1 to S10.

ASM does not own the copyrights to Supplemental Material that may be linked to, or accessed through, an article. The authors have granted ASM a non-exclusive, world-wide license to publish the Supplemental Material files. Please contact the corresponding author directly for reuse.
