# Supplementary material for: HIV DNA and transcription before and after ART in natural controllers compared to noncontrollers
Source: J Virol. 2026 Aug 24;100(9):e00482-26. doi: 10.1128/jvi.00482-26 (PMC13595968; doi:10.1128/jvi.00482-26)
Supplement: Supplementary file 1 [file jvi.00482-26-s0002.docx]

**Supplementary Material:**

**Table S1: Demographic and Clinical Characteristics of Controllers at T1 (Pre-ART)**

| ID | Gender | Ethnicity | Protective or susceptible HLA allele? | Age  (yr) | CD4  (cells per µL) | CD8  (cells per µL) | CD4/CD8 | Minimum time since  diagnosis (yr) | Pre-ART VL^1^ range  (copies / mL) | Last pre-ART  VL | Controller  status |
| --- | --- | --- | --- | --- | --- | --- | --- | --- | --- | --- | --- |
| 1201 | Female | Black/  African American | Susceptible  (B*07:02) | 49 | 689 | 2234 | 0.31 | 12.8 | <LOD^2^ to 533 | <40 | Viremic |
| 1218 | Male | White/ European American | Protective  (B*27:05) | 59 | 681 | 1665 | 0.41 | 7.6 | 1632-2511 | 1815 | Viremic |
| 1349 | Male | White/  European American | Protective  (B*27:05)  Susceptible (B*35:01) | 54 | 598 | 798 | 0.75 | 5.8 | <LOD (2 blips to 106) | <40 | Elite |
| 1369 | Male | Mixed/  Multiracial | Protective  (B*13:02,  B*44:03) | 44 | 507 | 952 | 0.53 | 8.6 | <LOD to 322 | 60 | Viremic |
| 1507 | Male | White/  European American | ?  (B*27:0502 to 0506 or B*27:08) | 57 | 583 | 1409 | 0.41 | 24.3 | <LOD (1 blip to 85) | <40 | Elite |
| 1549 | Female | Black/  African American | Protective  (B*27:05,  B:42:01) | 61 | 1310 | 1128 | 1.16 | 29.9 | <LOD to 400 | 153 | Viremic |
| 1609 | Male | White/  European American | Susceptible  (B*51:01) | 50 | 427 | 697 | 0.61 | 22.1 | <LOD to 534 | 534 | Viremic |
| 1762 | Male | White/  European American | Protective  (B*27:05) | 50 | 566 | 496 | 1.14 | 0.5 | <LOD to 302 (1 blip) | <40 | Elite |
| 3706 | Male | White/  European American | Susceptible  (B*07:02) | 26 | 1321 | 943 | 1.40 | 0.3 | <LOD | <40 | Elite |
| 4014 | Male | Black/  African American | Protective  (B*57:02) | 77 | 719 | 763 | 0.94 | 32.6 | <LOD (1 blip to 54) | <40 | Elite |
| Median |  |  |  | 52 | 640 | 948 | 0.68 | 10.7 |  | <40 |  |

^1^VL=viral load (plasma HIV RNA)

^2^LOD=Approved limit of detection of the clinical assay

**Table S2: Demographic and Clinical Characteristics of Controllers at T2-T3**

| ID | Time | Age  (yr) | CD4  (cells per µL) | CD8  (cells per µL) | CD4/CD8 | Last VL  (copies per mL) | Time on ART  (yr) | ART regimen^1^ |
| --- | --- | --- | --- | --- | --- | --- | --- | --- |
| 1201 | T2 | 56 | 673 | 1385 | 0.49 | N/A | 0.58 | EGV/TDF/FTC/COBI |
| 1218 | T2 | 61 | 708 | 801 | 0.88 | N/A | 1.68 | RGV, 3TC/ABC |
| 1218 | T3 | 63 | 1042 | 788 | 1.32 | N/A | 4.47 | RGV, 3TC/ABC |
| 1349 | T2 | 55 | 824 | 786 | 1.05 | <40 | 0.47 | RGV, FTC/TDF |
| 1369 | T2 | 48 | 539 | 939 | 0.57 | N/A | 1.55 | EGV/TDF/FTC/COBI |
| 1369 | T3 | 51 | 601 | 792 | 0.76 | <40 | 4.74 | EGV/TAF/FTC/COBI |
| 1507 | T2 | 59 | 691 | 1503 | 0.46 | <40 | 1.00 | RGV, FTC/TDF |
| 1549 | T2 | 65 | 1265 | 1302 | 0.97 | N/A | 2.75 | RPV/TAF/FTC |
| 1549 | T3 | 66 | 1290 | 980 | 1.32 | N/A | 3.83 | RPV/TAF/FTC |
| 1609 | T2 | 51 | 601 | 843 | 0.71 | N/A | 0.63 | RGV, FTC/TDF |
| 1609 | T3 | 55 | 549 | 497 | 1.10 | N/A | 4.53 | DTG, FTC/TDF |
| 1762 | T2 | 54 | 976 | 973 | 1.00 | <20 | 3.68 | RGV, FTC/TDF |
| 1762 | T3 | 55 | 852 | 765 | 1.11 | <20 | 4.65 | EGV/TDF/FTC/COBI |
| 3706 | T2 | 27 | 1952 | 1069 | 1.83 | <40 | 1.25 | RPV/TDF/FTC |
| 3706 | T3 | 29 | 1213 | 942 | 1.29 | <40 | 2.75 | RPV/TAF/FTC |
| 4014 | T2 | 83 | 721 | 609 | 1.18 | <40 | 2.75 | RPV/TAF/FTC |
| 4014 | T3 | 85 | 611 | 437 | 1.40 | <30 | 4.78 | RPV/TAF/FTC |
| Median at T2 |  | 56 | 715 | 956 | 0.93 |  | 1.40 | 9/10 TF, 1/10 ABC  7/10 INSTI, 3/10 RPV |
| Median at T3 |  | 55 | 952 | 788 | 1.29 |  | 4.53 | 6/7 TF, 1/7 ABC  4/7 INSTI, 3/7 RPV |

^1^ ART abbreviations: ABC=abacavir; COBI=cobicistat; DTG=dolutegravir; EGV=elvitegravir; FTC=emtricitabine; INSTI=integrase strand transfer inhibitor; RGV=raltegravir; RPV=rilpivirine; TF=tenofovir; TAF=tenofovir alafenamide; TDF=tenofovir disoproxil fumarate

**Table S3: Demographic and Clinical Characteristics in Controllers vs. ART-suppressed Noncontrollers**

| Comparison | HIV DNA regions and  HIV RNA per DNA |  |  | HIV RNA and RNA/RNA ratios |  |  | Defective and intact proviruses (IPDA) |  |  |
| --- | --- | --- | --- | --- | --- | --- | --- | --- | --- |
| Group | Controllers | NC^1^ | P value^2^ | Controllers | NC^1^ | P value^2^ | Controllers | NC^1^ | P value^2^ |
| Number of samples | 10 | 23 |  | 10 | 25 (except Pol) |  | 9 | 19 |  |
| Age (yrs)  Median (range)^3^ | 52 (26-77) | 50 (24-70) | 0.96 | 52 (26-77) | 54 (24-78) | 0.79 | 50 (26-77) | 48 (24-72) | 0.33 |
| Proportion male | 0.8 | 1.0 | 0.09 | 0.8 | 1.0 | 0.08 | 0.8 | 1.0 | 0.10 |
| CD4 (cells/uL)  Median (range)^3^ | 640 (427-1321) | 589 (132-1223) | 0.47 | 640 (427-1321 | 624 (132-1431) | 0.62 | 681 (427-1321) | 550 (387-1140) | 0.22 |
| CD8  (cells/uL)  Median (range)^3^ | 948 (496-2234) | 813 (333-2588) | 0.35 | 948 (496-2234) | 893 (333-2588) | 0.47 | 952 (496-2234) | 668 (301-2912) | 0.054 |
| CD4/CD8^3^ | 0.68 (0.31-1.40) | 0.75 (0.21-2.13) | 0.98 | 0.68 (0.31-1.40) | 0.75 (0.21-2.13) | 0.99 | 0.61 (0.31-1.40) | 0.89 (0.26-2.13) | 0.44 |
| Maximum recorded VL (copies/mL)  Median (range)^4^ | 312 (<40 to 2511) | 25700 (2408-415301) | <0.0001 | 312 (<40 to 2511) | 25700 (2408-415301) | <0.0001 | 322 (<40 to 2511) | 27769 (2408-415301) | <0.0001 |
| Time on ART^5^ | 1.4 (0.5-3.7) | 2.3 (0.23-9.8) | 0.16 | 1.4 (0.5-3.7) | 4.4 (0.23-16) | 0.10 | 1.6 (0.6-3.7) | 2.1 (0.23-23.1) | 0.38 |

^1^NC=Noncontrollers (ART-suppressed)

^2^Calculated using the Mann-Whitney test

^3^Age, CD4 counts, CD8 counts, and CD4/CD8 ratios are at T1 for controllers

^4^Data from noncontrollers are less commonly available from acute/early infection, so the available data may underestimate the peak for noncontrollers

^5^Time on ART refers to T2 for controllers

**Fig. S1: HIV DNA and RNA in “elite” controllers before ART and comparison to noncontrollers on suppressive ART.** HIV levels and ratios were compared between the subset of 5 elite controllers (inverted triangles) at T1 and ART-suppressed noncontrollers from two prior studies (circles or hexagons). Shown are comparisons in: A) HIV DNA; B) HIV proviruses; C) Total cell-associated HIV RNA; D) Ratio of HIV RNA to the corresponding HIV DNA region; and E) Ratios of one HIV transcript to another, indicating progression through the stages of elongation (elongated/initiated), completion (completed/elongated), and multiple splicing (multiply spliced/completed). Horizontal lines indicate medians; different colors and symbols indicate individual study participants. P-values (two-tailed) were calculated using the Mann-Whitney test.

**Fig. S2: HIV DNA and RNA in “long-term” controllers before ART and comparison to noncontrollers on suppressive ART.** HIV levels and ratios were compared between the subset of 8 individuals with more than 5 years of viral control at T1 (triangles denote viremic controllers; inverted triangles denote elite controllers) and ART-suppressed noncontrollers from two prior studies (circles or hexagons). Shown are comparisons in: A) HIV DNA; B) HIV proviruses; C) Total cell-associated HIV RNA; D) Ratio of HIV RNA to the corresponding HIV DNA region; and E) Ratios of one HIV transcript to another, indicating progression through the stages of elongation (elongated/initiated), completion (completed/elongated), and multiple splicing (multiply spliced/completed). Horizontal lines indicate medians; different colors and symbols indicate individual study participants. P-values (two-tailed) were calculated using the Mann-Whitney test.

**Fig. S3: HIV DNA and transcription in controllers at T1 compared to ART-suppressed noncontrollers.** Levels of HIV DNA (A, B), HIV RNA (C), HIV RNA per corresponding HIV DNA (D), or progression through stages of HIV transcription (E) were compared between controllers (triangles denote viremic controllers; inverted triangles denote elite controllers) and ART-suppressed noncontrollers measured in one prior study (circles). Horizontal lines indicate medians; different colors and shapes indicate individual study participants. P-values (two-tailed) were calculated using the Mann-Whitney test.

**Fig. S4: Changes in CD4 counts and CD4:CD8 ratios in elite controllers after the start of ART.** Absolute CD4+ T cell counts (A) and the ratio of CD4+ to CD8+ T cells (B) were measured in blood from the subset of 5 elite controllers (inverted triangles) before ART (T1) and after the start of ART (T2) using clinical assays. Horizontal lines indicate medians; different colors indicate individual study participants. P-values (two-tailed) were calculated using the Wilcoxon matched-pairs signed rank test.

**Fig. S5: Changes in CD4 counts and CD4:CD8 ratios in long-term controllers after the start of ART.** Absolute CD4+ T cell counts (A) and the ratio of CD4+ to CD8+ T cells (B) were measured in blood from the subset of 8 long-term controllers (triangles denote viremic controllers; inverted triangles denote elite controllers) before ART (T1) and after the start of ART (T2, T3) using clinical assays. Horizontal lines indicate medians; different colors indicate individual study participants. P-values (two-tailed) were calculated using the Wilcoxon matched-pairs signed rank test.

**Fig. S6: Changes in HIV DNA and HIV transcription in elite controllers after the start of ART.** HIV DNA and HIV transcription were measured in blood from the subset of 5 elite controllers (inverted triangles) before ART (T1) and after ART (T2). Shown are: A. Levels of the TAR, R-U5-pre-gag, pol, and U3-U5 HIV DNA regions; B. Levels of 3’ defective (Psi+RRE-), 5’ defective (Psi-RRE+), and intact (Psi+RRE+) HIV proviruses; C. Levels of initiated (TAR), 5’ elongated (R-U5-pre-gag), mid-transcribed/unspliced (pol), completed (U3-polyA), and multiply spliced (tat-rev) HIV transcripts; and D. Ratios of initiated (TAR), 5’ elongated (R-U5-pre-gag), mid-transcribed/unspliced (pol), and completed (U3-polyA) HIV RNA to the corresponding HIV DNA regions (average level of each HIV transcript per provirus. Horizontal lines indicate medians; different colors indicate individual study participants. P-values (two-tailed) were calculated using the Wilcoxon matched-pairs signed rank test.

**Fig. S7: Changes in HIV DNA and HIV transcription in long-term controllers after the start of ART.** HIV DNA and HIV transcription were measured in blood from the subset of 8 long-term controllers (triangles denote viremic controllers; inverted triangles denote elite controllers) before ART (T1) and after ART (T2). Shown are: A. Levels of the TAR, R-U5-pre-gag, pol, and U3-U5 HIV DNA regions; B. Levels of 3’ defective (Psi+RRE-), 5’ defective (Psi-RRE+), and intact (Psi+RRE+) HIV proviruses; C. Levels of initiated (TAR), 5’ elongated (R-U5-pre-gag), mid-transcribed/unspliced (pol), completed (U3-polyA), and multiply spliced (tat-rev) HIV transcripts; and D. Ratios of initiated (TAR), 5’ elongated (R-U5-pre-gag), mid-transcribed/unspliced (pol), and completed (U3-polyA) HIV RNA to the corresponding HIV DNA regions (average level of each HIV transcript per provirus. Horizontal lines indicate medians; different colors indicate individual study participants. P-values (two-tailed) were calculated using the Wilcoxon matched-pairs signed rank test.

**Fig. S8: Changes in HIV DNA and HIV transcription in controllers after the start of ART.**

HIV DNA and HIV transcription were measured in blood from viremic controllers (triangles) and elite controllers (inverted triangles) before ART (T1) and a median of 1.4 years (T2) and 4.5 yrs (T3) after the start of ART, including: A. Levels of the TAR, R-U5-pre-gag, pol, and U3-U5 HIV DNA regions; B. Levels of 3’ defective (Psi+RRE-), 5’ defective (Psi-RRE+), and intact (Psi+RRE+) HIV proviruses; C. Levels of initiated (TAR), 5’ elongated (R-U5-pre-gag), mid-transcribed/unspliced (pol), completed (U3-polyA), and multiply spliced (tat-rev) HIV transcripts; and D. Progression through stages of HIV transcriptional elongation (elongated/initiated RNA), completion (completed/elongated RNA), and multiple splicing (multiply spliced/completed RNA). Horizontal lines indicate medians; different colors indicate individual study participants. P-values (two-tailed) were calculated using the Wilcoxon matched-pairs signed rank test.

**Fig. S9: Changes in HIV RNA per corresponding DNA in controllers after the start of ART.** HIV DNA and HIV RNA were measured in viremic controllers (triangles) and elite controllers (inverted triangles) before ART (T1) and a median of 1.4 years (T2) and 4.5 yrs (T3) after the start of ART. To account for differences in infection frequency and/or proviral mutations, levels of initiated, 5’ elongated, mid-transcribed (pol), and completed HIV RNA in controllers were normalized to levels of the same or corresponding HIV DNA region measured in the same cells at the same timepoint. Horizontal lines indicate medians; different colors indicate individual study participants. P-values (two-tailed) were calculated using the Wilcoxon matched-pairs signed rank test.

**Fig. S10: HIV DNA and transcription in ART-suppressed controllers compared to ART-suppressed noncontrollers with similar time on ART.** Levels of HIV DNA (A, B), HIV RNA (C), HIV RNA per corresponding HIV DNA (D), or progression through stages of HIV transcription (E) were compared between ART-treated controllers at T2 (triangles denote controllers who were viremic before ART; inverted triangles denote elite controllers) and a subset of ART-suppressed noncontrollers (circles or hexagons) with similar duration of ART (median=1.0 year). Horizontal lines indicate medians; different colors and shapes indicate individual study participants. P-values (two-tailed) were calculated using the Mann-Whitney test.
